# Phenylketonuria and Gut Microbiota: A Controlled Study Based on Next-Generation Sequencing

**DOI:** 10.1371/journal.pone.0157513

**Published:** 2016-06-23

**Authors:** Felipe Pinheiro de Oliveira, Roberta Hack Mendes, Priscila Thiago Dobbler, Volker Mai, Victor Salter Pylro, Sheldon G Waugh, Filippo Vairo, Lilia Farret Refosco, Luiz Fernando Würdig Roesch, Ida Vanessa Doederlein Schwartz

**Affiliations:** 1 Programa de Pós-Graduação em Genética e Biologia Molecular, Universidade Federal do Rio Grande do Sul, Porto Alegre, Rio Grande do Sul, Brazil; 2 Serviço de Genética Médica, Hospital de Clínicas de Porto Alegre, Porto Alegre, Rio Grande do Sul, Brazil; 3 Laboratório *Basic Research and Advanced Investigations in Neurosciences* (B.R.A.I.N)—Centro de Pesquisa Experimental, Hospital de Clínicas de Porto Alegre, Porto Alegre, Rio Grande do Sul, Brazil; 4 Centro Interdisciplinar de Pesquisas em Biotecnologia–CIP-Biotec, Campus São Gabriel, Universidade Federal do Pampa, São Gabriel, Rio Grande do Sul, Brazil; 5 Department of Epidemiology, College of Public Health and Health Professions and College of Medicine, Emerging Pathogens Institute, University of Florida, Gainesville, Florida, United States of America; 6 Genomics and Computational Biology Group, René Rachou Research Centre–CPqRR, Belo Horizonte, Minas Gerais, Brazil; University of Illinois at Urbana-Champaign, UNITED STATES

## Abstract

Phenylketonuria (PKU) is an inborn error of metabolism associated with high blood levels of phenylalanine (Phe). A Phe-restricted diet supplemented with L-amino acids is the main treatment strategy for this disease; if started early, most neurological abnormalities can be prevented. The healthy human gut contains trillions of commensal bacteria, often referred to as the gut microbiota. The composition of the gut microbiota is known to be modulated by environmental factors, including diet. In this study, we compared the gut microbiota of 8 PKU patients on Phe-restricted dietary treatment with that of 10 healthy individuals. The microbiota were characterized by 16S rRNA sequencing using the Ion Torrent™ platform. The most dominant phyla detected in both groups were Bacteroidetes and Firmicutes. PKU patients showed reduced abundance of the Clostridiaceae, Erysipelotrichaceae, and Lachnospiraceae families, Clostridiales class, *Coprococcus*, *Dorea*, *Lachnospira*, *Odoribacter*, *Ruminococcus* and *Veillonella* genera, and enrichment of *Prevotella*, *Akkermansia*, and Peptostreptococcaceae. Microbial function prediction suggested significant differences in starch/glucose and amino acid metabolism between PKU patients and controls. Together, our results suggest the presence of distinct taxonomic groups within the gut microbiome of PKU patients, which may be modulated by their plasma Phe concentration. Whether our findings represent an effect of the disease itself, or a consequence of the modified diet is unclear.

## Introduction

Phenylketonuria (PKU, OMIM 261600) is an autosomal recessive disorder characterized by high plasma levels of phenylalanine (Phe) due to pathogenic variations in the *PAH* gene, which encodes phenylalanine hydroxylase (PAH, EC 1.14.16.1). PAH catalyzes the hepatic conversion of Phe to tyrosine (Tyr), using tetrahydrobiopterin (BH4) as a co-factor [1]. In Brazil, the prevalence of PKU has been estimated at 1:24,780 [2]. There is significant clinical heterogeneity within PKU patients, even within the same family [3], suggesting an effect of environmental factors. Treatment for this disease is based on restriction of Phe intake and supplementation of essential amino acids and micronutrients, and/or on the administration of 6*R*-BH4 (sapropterin) to BH4-responsive patients [4]. Other strategies may include the use of large neutral amino acids (LNAA) and of glycomacropeptide [5]. To achieve normal physical and neurocognitive development, treatment should be started as early as possible [6]. Untreated patients usually develop mental retardation, seizures, and autism-like symptoms, among other neurological findings.

Various external factors, including antibiotic exposure and differences in dietary pattern, can modify the composition of the gut microbiota. Byproducts of microbial metabolism can interact with the host metabolism to change intestinal function locally, but also distally, acting on liver, brain, muscle, and adipose tissues [7]. Changes in the composition and diversity of the microbiota may result in an imbalance that favors colonization with potentially detrimental bacteria, a phenomenon known as dysbiosis. This condition has been related to autoimmunity in patients with type 1 diabetes and to obesity [8–10]. Microbiota distortions have also been found to correlate with several inflammatory bowel diseases, such as Crohn’s disease and ulcerative colitis [11].

The nutritional composition of PKU diets might change gut microbial ecology and affect host physiology. This study aims to characterize the gut microbiota of PKU patients using next-generation sequencing (NGS) technology and to compare its composition with that of healthy individuals.

## Material and Methods

### Experiment design

This observational, cross-sectional study used a convenience sampling strategy. The study protocol was approved by the ethics committee of Hospital de Clínicas de Porto Alegre (HCPA), Brazil, and all patients and healthy controls and/or their legal guardians provided written informed consent. Patients were recruited from the PKU Clinics of the Medical Genetics Service (MGS), HCPA, Rio Grande do Sul, Brazil, and healthy controls were recruited among the non-PKU population of Rio Grande do Sul through invitations. The inclusion criteria for patients were: a) established diagnosis of PKU; b) current dietary treatment; and c) no other chronic disease. Controls could not be related to PKU cases; could not have any chronic or acute disease; and could not have any neurological abnormality. For 8 controls, we were able to confirm that a diagnosis of PKU had also been excluded by newborn screening.

Clinical parameters such as current age, plasma Phe and Tyr levels, and daily Phe intake were obtained by reviewing medical records. Measurement of plasma Phe and Tyr levels was performed by HPLC at MGS-HCPA. Patients and controls completed a specific questionnaire including questions on comorbidities, use of medicines, and dietary intake.

Participants received a stool collection kit consisting of a Styrofoam box containing a sterile container to store the stool sample, a gel ice pack, a sterilized spatula to collect the stool sample, and printed instructions for stool collection. All participants were instructed to collect stool in their own homes one day before their medical appointments at HCPA. The stool sample was kept at -20°C (household freezer) and delivered the next day, on ice, during the scheduled appointment. Samples were obtained from 8 PKU patients and 10 controls.

### Dietary intake analysis, anthropometric evaluation, and statistical analysis

Patients and controls completed a 24-h dietary recall questionnaire [12] during a face-to-face interview with a dietitian, which took place on the same day the stool sample was delivered. Macronutrient (carbohydrates, proteins, and lipids), amino acid and mineral intakes were analyzed using the Nutribase™ software.

Anthropometric assessment consisted of weight (kg) and height (cm) measurements. Body weight was measured using calibrated digital scales. Height was measured with a wall-mounted stadiometer with 1-mm resolution. Anthropometric measurements were recorded and classifications for age and sex performed using the World Health Organization AnthroPlus software suite. The variables of interest were BMI-for-age Z-scores.

Numeric variables were compared using the Mann-Whitney *U* test, and summarized as means ± SEM. Categorical variables were compared using Fisher’s exact test (*p* ≤ 0.05).

### Microbial DNA extraction and 16S rRNA gene amplification

Microbial DNA was isolated from 200–300 mg fecal samples using the QIAamp Fast DNA Stool Mini Kit (Qiagen, Valencia, CA), following manufacturer instructions. DNA quality was determined by spectrophotometry using a NanoVue™ system (GE Healthcare). All DNA samples were stored at -20°C until use for PCR reactions. Gut microbial community determination was based on partial 16S rRNA gene (V4 region) sequences, directly amplified using bacterial/archaeal primer 515F/806R [13]. PCR reactions were carried out with the Platinum® *Taq* DNA Polymerase High Fidelity kit (Invitrogen). The mixtures contained 5 μl of 10X High Fidelity PCR buffer, 50 mM MgSO_4_, 10 mM dNTP Mix, 100 mM of each primer, 1 U of *Taq* polymerase, and approximately 100 ng of DNA template in a final volume of 50 μL. The PCR conditions were 94°C for 5 minutes, 30 cycles of 94°C for 45 s, 56°C for 45 s, and 68°C for 1 minute, followed by a final extension step of 68°C for 10 minutes. The PCR products were purified with AMPure XP PCR Purification Kit (Agencourt), quantified with a Qubit Fluorometer (Invitrogen), and combined in equimolar ratios. The 16S rRNA gene fragments were sequenced using the Ion Torrent™ platform for unidirectional sequencing of the amplicon libraries. Barcoded primers were used to multiplex the amplicon pools. Twelve-base barcode sequences were added to the 5′-end of the reverse primers using the error-correcting barcode method [14]. A two-base linker sequence was inserted between the adapter and the 16S rRNA primers to reduce any effect the composite primer on PCR efficiency. The Ion OneTouch™ 2 System and Ion PGM™ Template OT2 400 Kit were used for library preparation. Finally, to obtain high-sequence coverage and detect rare microbes inhabiting the samples, up to 15 samples chosen at random were loaded on an Ion 314™ Chip v2. A total of three runs were performed within this study. Raw sequences were deposited in the NCBI Sequence Read Archive under experiment number SRX1044553.

### 16S rRNA read processing for downstream analyses

The 16S rRNA reads were analyzed following the recommendations of the Brazilian Microbiome Project [15], for efficient removal of sequencing artifacts that might exacerbate biases via the presence of chimeric sequences and sequence errors. Briefly, an Operational Taxonomic Unit (OTU) table was built using the UPARSE pipeline [16]. The reads were truncated at 200 bp and quality-filtered using a maximum expected error value of 0.5, meaning that, on average, one nucleotide in every two sequences is incorrect. Filtered reads were dereplicated and singletons were removed. These sequences were clustered into OTUs at a 97% similarity cutoff following the UPARSE pipeline. After clustering, the sequences were aligned and taxonomically classified using the Greengenes reference database (version 13.8). Finally, to lower the effects of variation in gut microbiome that were observed between individuals, OTUs with low representativeness (those present in less than 30% of patients) were identified and removed.

### Library comparisons

To evaluate whether the sampling effort was appropriate to detect the majority of the gut bacterial community in each patient, sequence coverage was calculated at a 97% similarity cutoff using Good’s coverage [17].

For overall comparison of significant differences among bacterial communities from PKU patients and controls, principal coordinates analysis (PCoA) was performed within the R environment [18] using the phyloseq package [19]. The OTU matrix was rarefied at 1,000 sequences per sample and, for each pair of environments, a dissimilarity matrix was calculated using Bray-Curtis and binary distance metrics. Adonis implemented in vegan package [20] was used to test whether differences among groups of samples observed by PCoA were significant. For estimation of alpha diversity, the data set was rarefied to the same number of sequences (1,000 sequences per sample) [21] and the observed OTU richness, Chao 1 and ACE richness estimators, Shannon, Simpson, and inverse Simpson diversity indexes were calculated and further plotted using the phyloseq package [19].

The Statistical Analysis of Metagenomic Profiles (STAMP) v2 software package [22] was used to determine differences in the relative abundances of categories (i.e., taxa) between treatments. Differences between treatments were evaluated using White’s non-parametric *t*-test, while confidence intervals were calculated using the bootstrap method. Taxonomic units with a difference between proportions below 1% were excluded from analysis.

### Metagenome prediction

To predict functions performed by different microbial groups found within control and PKU patients, the 16S rRNA dataset was input into the Phylogenetic Investigation of Communities by Reconstruction of Unobserved States (PICRUSt) pipeline [23]. The outputted OTU table was normalized according to the 16S rRNA gene copy number per genome and a final metagenome functional prediction was created. To determine statistical differences between the control and PKU metagenomes, the STAMP software package was used [22]. Statistical hypothesis testing was performed using Welch’s *t*-test, while confidence intervals were calculated using Welch’s inverted method with Bonferroni correction.

## Results

The characteristics of the 8 unrelated PKU patients and 10 healthy individuals are shown in Table 1 and S1 Table. The mean plasma Phe level among PKU patients was 307.33 μmol/L (SEM = 66.58). All were on a Phe-restricted diet supplemented with a metabolic formula (protein substitute) without prebiotics (S1 Table). Only one patient was being breastfed, none was receiving BH4-, LNAA- or glycomacropeptide-based treatments, two reported the use of oral antibiotics in the previous 6 months (none in the week preceding stool sample collection), and four had taken other drugs in the week preceding stool sample collection (ferrous sulphate, vitamin A and D, and prednisolone). Among controls, one was being breastfed (S1 Table), three reported the use of oral antibiotics in the previous 6 months (none in the week preceding stool sample collection), and none reported the use of other oral drugs in the week preceding stool sample collection.

**Table 1:** Comparison of phenylketonuria and control groups.

Variables	PKU (n = 8)	Controls (n = 10)	*p-*value
Age (years)	4.24 ± 1.74	6.06 ± 1.78	0.459
Sex (male: female)	6:2	4:6	0.188
Body weight (kg)	21.02 ± 6.32	25.00 ± 5.95	0.423
Height (cm)	97 ± 11.03	115 ± 10.04	0.197
BMI (kg/m²)	18.48 ± 1.30	16.87 ± 1.55	0.168
Type of birth (natural: cesarean)	3:5	2:8	0.607
Antibiotic therapy* (yes: no)	2:6	3:7	0.788
Pets at home (yes: no)	6:2	6:4	0.638
Daily intake			
-calories (kcal)	1227.92 ± 187.91	1277.56 ± 78.43	0.929
-protein (kcal)	316.50 ± 61.64	291.30 ± 20.86	0.859
-carbohydrate (kcal)	1009.90 ± 177.6	636.50 ± 37.80	**0.013**
-lipids (kcal)	145.08 ± 30.16	339.75 ± 40.09	**0.003**
-protein (g)**	79.12 ± 15.41	72.82 ± 15.21	0.859
-carbohydrate (g)	215.08 ± 39.65	159.11 ± 9.44	0.131
-starch (g)	12.77 ± 9.75	15.28 ± 7.41	0.532
-total simple sugars (g)	66.20 ± 19.02	56.61 ± 4.76	0.722
-glucose (g)	6.29 ± 2.70	3.90± 0.81	0.721
-fructose (g)	8.05 ± 3.28	4.19 ± 0.69	0.592
-fibers (g)	17.44 ± 3.42	12.37± 0.97	0.076
-lipids (g)	16.12 ± 3.35	37.75 ± 4.45	**0.003**
-saturated fat (g)	2.20 ± 0.85	15.69 ± 1.96	**0.001**
-monounsaturated fat (g)	3.02 ± 1.44	9.08 ± 1.55	**0.001**
-polyunsaturated fat (g)	2.27 ± 0.82	4.36 ± 1.90	0.286
-cholesterol (mg)	5.15 ± 4.16	162.60 ±15.89	**0.001**

PKU, phenylketonuria.

* in the previous 6 months.

** The protein substitute corresponded to 73% (range = 47.42–95.29) of the daily protein intake for patients.

Numeric variables were summarized as means ± SEM and compared using the Mann-Whitney *U* test. Categorical variables were compared using Fisher’s exact test. Significant *p*-values (< 0.05) highlighted in bold.

Information based on the dietary recalls showed large differences among patients and controls. As expected, most of the caloric intake of PKU patients came from carbohydrates (about 80%, Table 1), and most of their protein intake came from the protein substitute (Table 1 and S1 Table). PKU diet tended to be rich in amino acids from the protein substitute and poor in phenylalanine (S2 Table). On the other hand, individuals from the control group had a higher intake of lipids and natural amino acids (Table 1 and S2 Table). Regarding minerals, the intake of selenium was low in PKU, while calcium, phosphorus, iron, manganese and zinc were high and mainly supplied by the protein substitute (S3 Table).

### Microbiota composition

**Fig 1:**
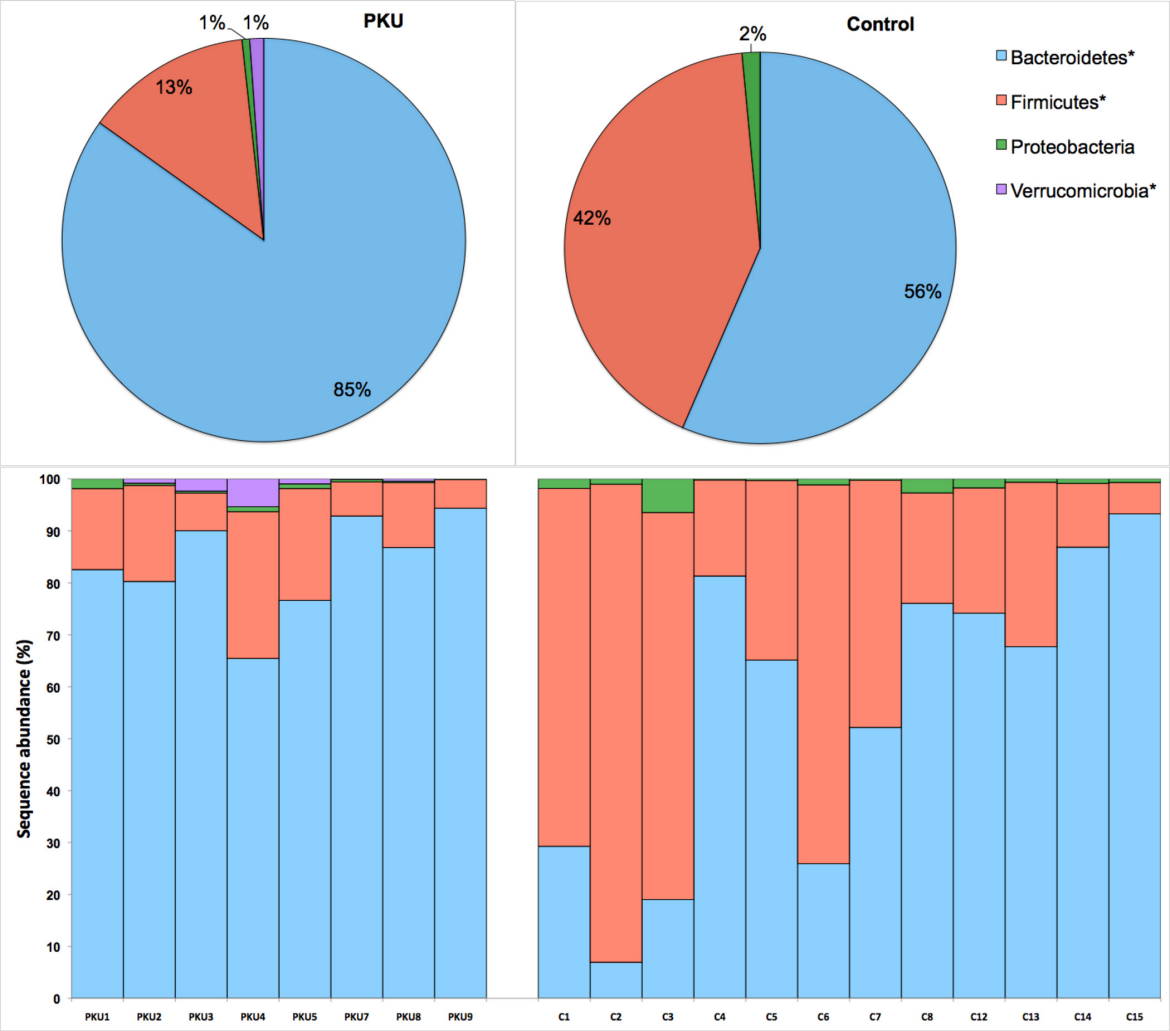
Frequencies of most abundant phyla (>1% of group sequences) in the phenylketonuria (n = 8) and control (n = 10) groups. *p-value < 0.05. Top panel represents the average abundance per treatment. Bottom panel represents the average of phyla per patient.

**Fig 2:**
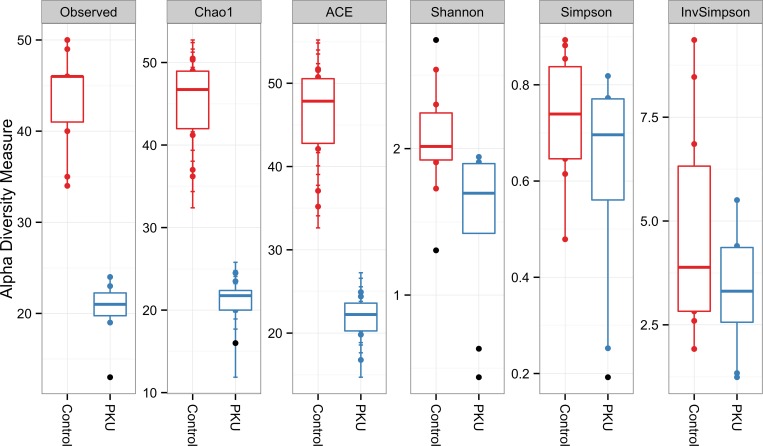
Alpha diversity measurements of microbial communities in the phenylketonuria and control groups. Each panel represent one alpha diversity measure as follow: Observed = total number of OTU’s observed; Chao1 and ACE = richness estimators (estimate the total number of OTU’s present in a community); Shannon, Simpson and InvSimpson = microbial indexes of diversity. Boxes span the first to third quartiles; the horizontal line inside the boxes represents the median. Whiskers extending vertically from the boxes indicate variability outside the upper and lower quartiles, and the single black circles indicate outliers.

After filtering reads by removing sequencing artifacts, a total of 106,035 high-quality sequences longer than 200 bp, with an average of 5,890 sequences/sample, were retained. Good’s coverage >99% (S1 Table) indicated that the dataset was representative of the bacterial communities present and allowed comparison of alpha and beta diversity measures. A total of 10 known bacterial phyla were detected, the most dominant being Bacteroidetes, Firmicutes, Proteobacteria, and Verrucomicrobia. The least abundant phyla (less than 1%) were Actinobacteria, Elusimicrobia, Fusobacteria, Lentisphaerae, Synergistetes, and Tenericutes. Among the dominant phyla, Bacteroidetes and Verrucomicrobia were more and Firmicutes less prevalent in PKU samples (Fig 1). Alpha diversity measures also indicated a lower diversity in PKU patients compared to controls (Fig 2).

**Fig 3:**
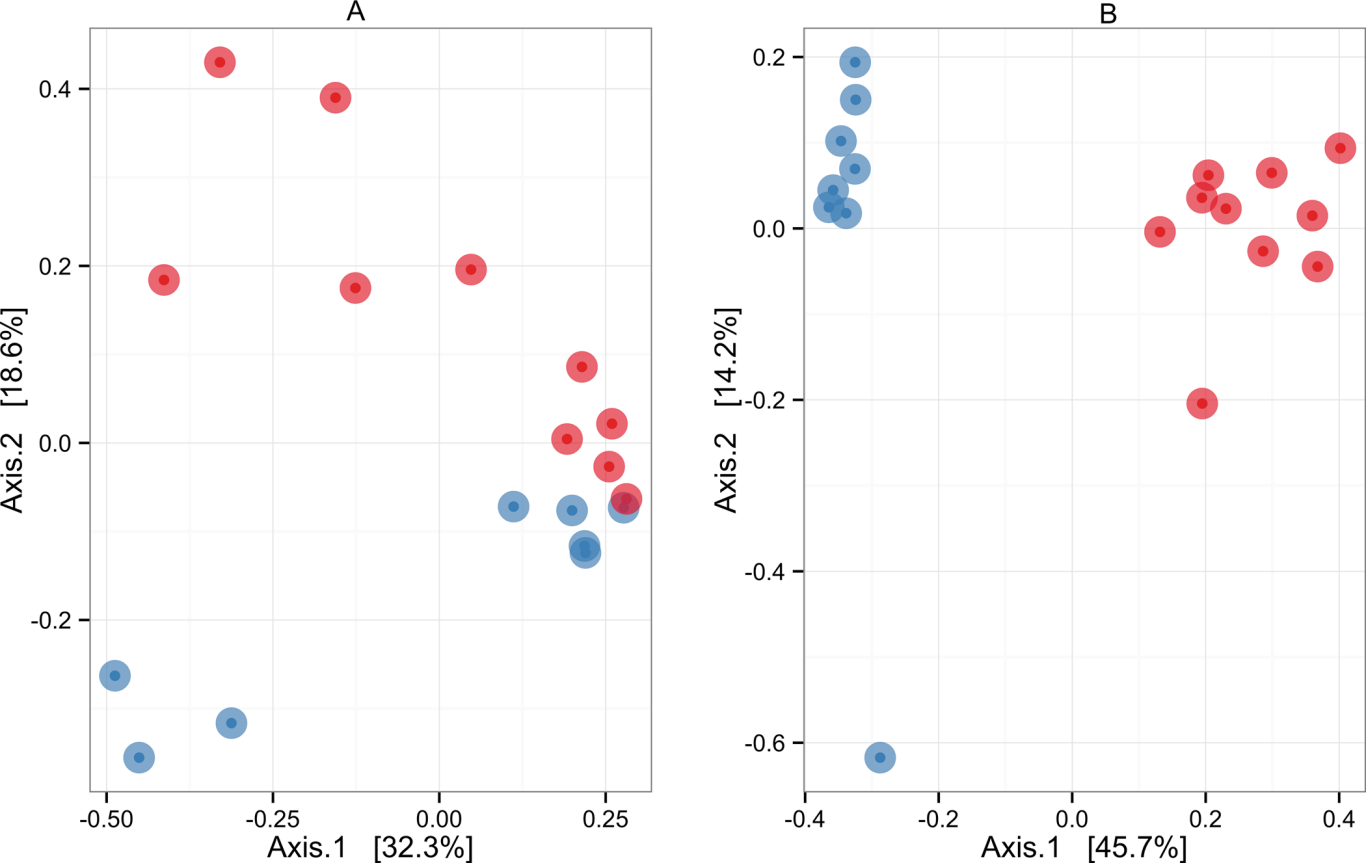
Overall comparisons of microbial communities based on principal coordinates analysis (PCoA), depicting clusters of bacterial communities in 18 samples each from the phenylketonuria (blue) and control (red) groups. (A) Bray-Curtis distance metrics. (B) Binary distance metrics. Each point represents a microbial community. Points closer to each other represent similar microbial communities, while points farther from each other represent dissimilar microbial communities. The statistical significance of sample groupings (control × PKU) was tested with the Adonis function using distance matrices as primary input. *R*^2^ values were 0.116 (*p* = 0.034) for Bray-Curtis distance metrics and 0.437 (*p* = 0.001) for binary distance metrics.

Ordination analysis using a binary distance matrix showed that the microbial communities from the control group were quite different from those of the PKU group (Fig 3A). When the relative abundance of taxa was taken into account (Bray-Curtis distance) (Fig 3B), the microbial communities remained distinct, although closer to each other. The difference between groups was confirmed by Adonis analysis carried out using either binary or Bray-Curtis metrics. The *R*^2^ value (effect size) was 0.437 (*p* = 0.001) for binary distance and 0.116 (*p* = 0.034) for Bray-Curtis distance.

White’s non-parametric *t*-test was used to determine phylotypes that were statistically different between cases and controls (Table 2). Many bacterial taxa were reduced in the PKU group. Most significant differences were observed for members of the Clostridiaceae, Erysipelotrichaceae and Lachnospiraceae family, Clostridiales class, *Coprococcus*, *Dorea*, *Lachnospira*, *Odoribacter*, *Ruminococcus* and *Veillonella* genera, which were enriched in the control group. Furthermore, three phylotypes belonging to the genera *Akkermansia* and *Prevotella* and to the Peptostreptococcaceae family were enriched in the PKU group.

**Table 2:** Bacterial phylotypes that differed between controls and patients with phenylketonuria, based on White’s non-parametric *t*-test.

Bacterial phylotypes	Control		PKU		*p*-values
	mean	std. dev.	mean	std. dev.	
	--------------- % ---------------				
*Akkermansia*	0.00	0.00	1.45	1.99	0.004
*Clostridiaceae;g_*	2.71	4.72	0.00	0.00	0.004
*Clostridiales;f_;g_*	1.03	1.05	0.00	0.00	0.002
*Coprococcus*	1.77	1.42	0.00	0.00	0.002
*Dorea*	0.43	0.54	0.00	0.00	0.002
*Erysipelotrichaceae;g_*	0.92	1.63	0.00	0.00	0.002
*Lachnospira*	0.63	0.97	0.00	0.00	0.002
*Lachnospiraceae;g_*	14.60	19.17	0.95	1.71	0.002
*Odoribacter*	0.93	0.93	0.00	0.00	0.004
*Peptostreptococcaceae;g_*	0.00	0.00	0.56	1.05	0.004
*Prevotella*	0.00	0.00	16.63	30.33	0.002
*Rikenellaceae;g_*	0.31	0.34	0.00	0.00	0.014
*Ruminococcus*	1.97	1.84	0.00	0.00	0.004
*Veillonella*	0.49	1.27	0.00	0.00	0.004

### Metagenome prediction

**Fig 4:**
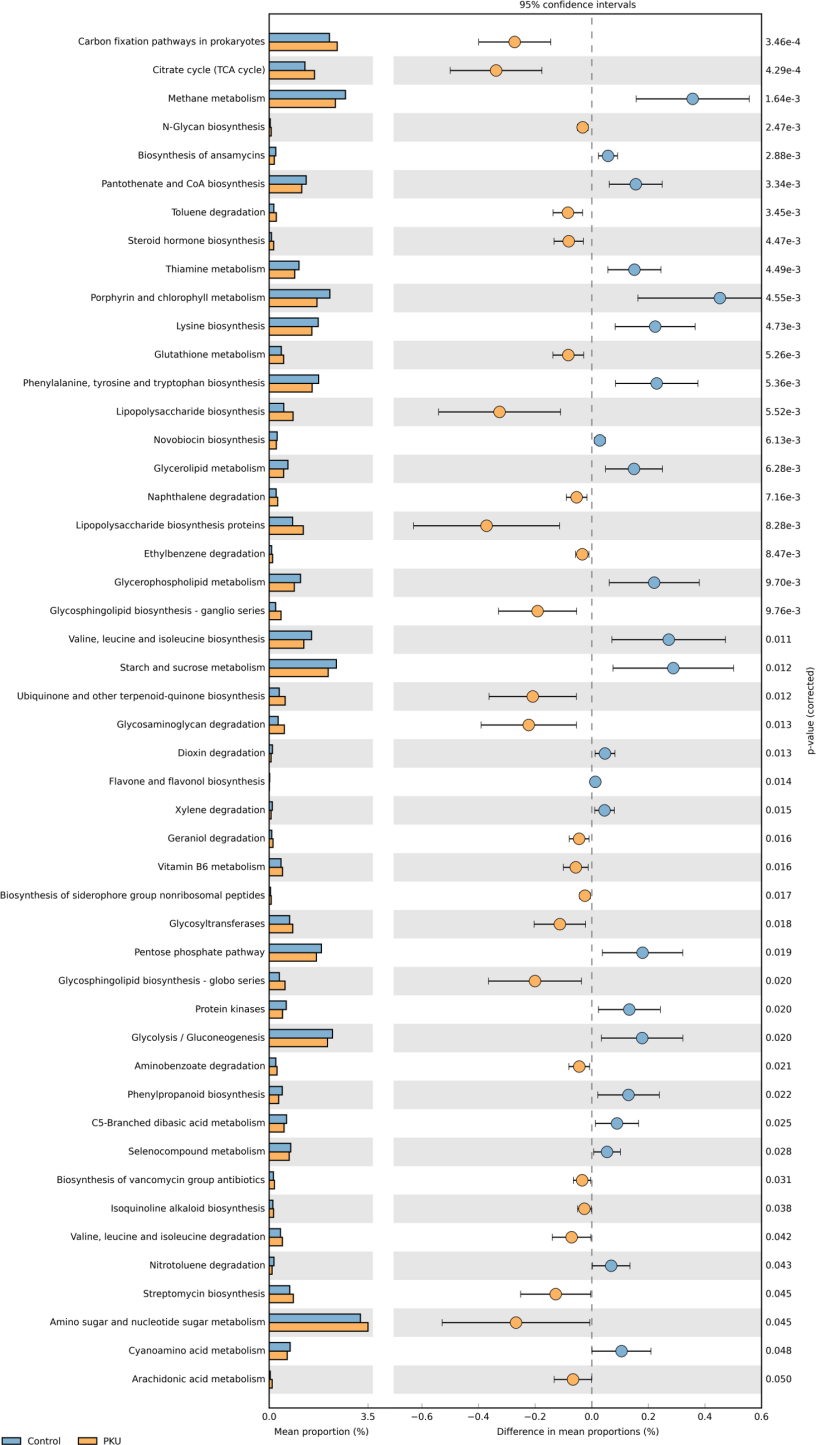
Relative abundance of predicted microbial genes related to metabolism in control (blue) and phenylketonuria (orange) samples, based on Welch’s *t*-test (*p* ≤ 0.05). The colored circles represent 95% confidence intervals calculated using Welch’s inverted method.

On the basis of PICRUSt analysis, 48 differentially abundant bacterial functions related to metabolism were observed between PKU and controls (*p* ≤ 0.05), with 23 of them being underrepresented in the PKU group (Fig 4), including those related to starch and sucrose metabolism, glycolysis/gluconeogenesis, and to Phe, Tyr, tryptophan, valine, leucine and isoleucine biosynthesis. Twenty-six were overrepresented in the PKU group (Fig 4), including those related to lipopolysaccharide (LPS) biosynthesis proteins and to glutathione metabolism.

## Discussion

To our knowledge, this is the first study to characterize the gut microbiota of PKU patients under dietary treatment using a NGS platform, the gold-standard method for this purpose. The only similar investigation is that by MacDonald *et al*. [24], who analyzed, through fluorescence in situ hybridization (FISH), selected bacterial groups (*bifidobacteria*, *lactobacilli–enterococci*, bacteroides distasonis/fragilis, *Clostridium histolyticum/lituseburense*, *E*. *rectale/C*.*coccoides* group and a subset of enterobacteriaceae) in the stool samples of 9 treated infants with PKU (median age = 7.86 weeks), aiming at evaluating the effect of a metabolic formula containing prebiotics. Before the inclusion, all 9 patients were receiving a protein substitute without prebiotics alone (n = 1) or in combination with breast milk (n = 3); with breast milk plus a protein substitute with prebiotics (n = 3); and with protein substitute with prebiotics (n = 2). Afterwards, all patients started to receive only the protein substitute with prebiotics. Stools were collected before and after the change in treatment, and no significant changes in *bifidobacteria* or *lactobacilli–enterococci* concentrations were observed (*bifidobacteria* corresponded to about 60% of total of bacterial count). In our study, the mean age of PKU patients was 4.24 years. According to Bäckhed *et al*. [25] the genera *Bifidobacterium*, *Lactobacillus*, *Collinsella*, *Granulicatella*, and *Veillonella* are considered as signatures of the 4-month microbiome. However, at 12 months a shift toward a more adult-like intestinal environment occurs and those microbes are no longer dominant. That explains why *bifidobacteria* was not detected in our study and indicates that due to differences in age, our study is not comparable with that of MacDonald *et al*. [24]. Also, direct comparisons between data obtained by different molecular methods—MacDonald *et al*. [24] used FISH and we use NGS—present restrictions due to the primers and reference database choice, DNA extraction methods, sequencing platform and bioinformatics approach.

Our data suggest that PKU patients have a different microbiota as compared to controls, a finding that could be associated with the clinical variability and different responses to treatment found among these individuals. Genetic and environmental factors might be related to microbial diversity and composition. Microbes in the human gut undergo selective pressures from environmental factors such as diet, antibiotics, probiotics, smoking, and drugs [26–29]. As expected, our sample of patients and controls differ regarding the daily intake of Phe, and, most probably, also regarding levels of Phe in plasma. But they also differ regarding many other aspects of the diet, such as the percentage of calories coming from carbohydrates and lipids, the percentage of proteins and amino acids coming from products of high biological value, and in the intake of selenium. Both groups did not differ regarding the use of antibiotics. So, the main modifiers acting in PKU patients are plasma Phe levels and diet, which consists of reduced Phe intake and supplementation with a protein substitute. Indeed, different plasma Phe levels among PKU patients appear to have contributed significantly to shifts in the gut microbiota. We have to point out, however, that only the patients’ group referred the use of ferrous sulfate, vitamins and prednisolone in the week preceding the stool sample collection. However the effect of such drugs on the gut microbiota is still unclear.

The host-associated gut microbiota, which is composed of around 10–100 trillion bacteria [30], contributes to maintaining human health by providing unique metabolic functions to the human host [31]. Functional maturation of the microbiota is thought to take place during the first three years of life [32], and plays an important role in allowing energy uptake from food fibers and complex carbohydrates that are not digested in the upper portion of the human digestive tract [33]. The gut microbiota plays another key role in stimulating and promoting maturation of the immune system and facilitating resistance against pathogens [34,35].

In this study, we collected stool samples from PKU patients and healthy controls using a safe, non-invasive method. Bacteroidetes and Firmicutes were the most dominant phyla found within the samples, corroborating prior findings in healthy humans [36,37] and the results obtained by Sawin *et al*. [38] in PKU mice. However, it should be noted that the biological significance of similarities at the phylum level is questionable, as many microbial species from the same phylum (e.g., Firmicutes) can be considered either pathogenic, e.g., *Staphylococcus aureus* [39], or probiotic, e.g., *Lactobacillus* spp. [40]. Moreover, marked differences at 97% nucleotide identity have been observed among individuals residing in the United States compared to healthy Amerindians from the Amazonas state of Venezuela and residents of rural Malawian communities [41]. Such geographic differences emphasize the need for local experiments to provide a better understanding of gut microbial ecology. When comparing the gut microbiota of healthy individuals, one of the most important findings was that each individual has a unique gut microbial composition [42].

The most significant differences in gut microbiota between patients and controls were observed for members of the Clostridiaceae, Erysipelotrichaceae, and Lachnospiraceae family, Clostridiales class, *Coprococcus*, *Dorea*, *Lachnospira*, *Odoribacter*, *Ruminococcus*, and *Veillonella* genera, which were enriched in the control group. Furthermore, three bacterial members—belonging to genera *Prevotella* and *Akkermansia* and to the *Peptostreptococcaceae* family—were enriched in the PKU group. The function of these organisms in the gut is unclear, but previous studies have demonstrated that *Akkermansia muciniphila* is present in low abundance in obese people and is inversely correlated with body weight in rodents and humans [43,44]. In our sample, however, there was no difference in weight or body mass index (BMI) between patients and controls. *Prevotella* was highly abundant in children who consumed diets with high amounts of plant polysaccharides and fiber, lower in animal protein and saturated fats [45], and was also abundant in our PKU group. In our study, patients and controls did not differ regarding total protein intake, but their protein intake patterns did differ qualitatively: PKU patients eat mainly protein of low biological value (e.g., those of vegetable origin and those provided by the metabolic formula), while the diet of controls, although composed of vegetables as well, contained animal sources of protein including beef, chicken, fish, milk, and dairy products. In healthy children, presence of the *Peptostreptococcaceae* family in the gut microbiota has been associated with household pets [46]; however, our sample of patients did not differ from controls regarding this variable.

According to metagenome prediction, the microbiome of PKU patients presented fewer genes involved in starch and sucrose metabolism and in glycolysis/gluconeogenesis. Overall, the PKU diet was higher in carbohydrate and lower in lipids (mainly monounsaturated and polyunsaturated fat) suggesting the dietary energy intake was associated with gut microbial activity. Such differences might reflect differential energy harvesting capacity [7]. In animal models of obesity and in obese human subjects, the microbiome is usually enriched with microbial genes involved in starch and glucose metabolism [7,10,30]. Differences in microbial starch and glucose metabolism might also affect production of short-chain fatty acids (SCFAs), which might suggest lower SCFA production by the gut microbiota of PKU patients. SCFAs (mainly acetate, propionate, and butyrate) have profound effects on gut health as an energy substrate for colonic epithelial and peripheral tissues, and act as modulators of inflammation [7]. Sawin *et al*. [37] studying nutritional management of PKU with prebiotic in mice model provided evidence that Glycomacropeptide increased concentrations of the SCFA acetate, propionate and butyrate as well as reduced cytokines and indices of inflammation. Those finding provided support for the concept that the unique PKU diet may be contributing to the dramatic change in the microbiome observed in this report.

The gut microbiome plays important roles in protein hydrolysis, providing free amino acids for both host and microbial metabolism functions, including amino acid biosynthesis [47]. PKU patients showed low abundance of microbial genes involved in biosynthesis of the amino acids phenylanine, tyrosine, tryptophan, valine, leucine, and isoleucine when compared with controls. The low level of microbial amino acid metabolism in the gut of PKU patients could be explained by their reduced Phe intake, by the restriction of natural protein intake [48], and by the use of PKU metabolic formulas, which are composed of amino acids such as tyrosine, tryptophan, isoleucine, leucine, and valine. Interestingly, Clayton [49] suggested an association between gut metabolism of Phe and autism, since some *Clostridium* species can convert Phe to 3-phenylpropanoic acid, with cinnamic acid as an intermediate. Cinnamic acid is a precursor of 3-(3-hydroxyphenyl)-3-hydroxypropionic acid (HPHPA), a potential biomarker for autism.

The number of genes involved in LPS biosynthesis was higher in the microbiome of PKU patients. The microbiome is a source of LPS, which may cause endotoxemia and inflammation in peripheral tissues [7,50]. LPS molecules bind to Toll-like receptor 4 (TLR4), which activates proinflammatory signaling cascades and expression of proinflammatory cytokines by gut epithelial cells [7]. This role of the microbiome in altering the host metabolism and modulating inflammation has already been demonstrated in type 2 diabetes mellitus, obesity, and the metabolic syndrome [7,50,51]. Although evidence of gut inflammation has not been found in PKU patients [52], treated PKU patients exhibit increased levels of proinflammatory cytokines in the blood [53], a finding which may suggest a role of the gut microbiome in promoting inflammation in PKU.

In addition, genes involved in glutathione metabolism were found in greater abundance in the microbiome of PKU patients. Glutathione is an antioxidant, and glutathione deficiency is associated with oxidative stress [54]. Changes in the microbiome associated with oxidative stress have been previously reported in animal models of chronic kidney disease [55]. Glutathione deficiency may provoke an imbalance between antioxidants and oxidants, generating oxidative stress, a situation that has been reported in patients with PKU [56–58]. While microbiome-associated changes in oxidative stress have already been described, the metabolic formula consumed by the PKU group contains antioxidants (vitamins A, C, D, E), suggesting that the microbiota might be modulated by the exogenous antioxidants provided by the formula.

In conclusion, our data indicate the presence of distinct taxonomic groups within the gut microbiome of PKU patients as compared with that of healthy controls. Whether the presence of these differentially abundant groups is a cause or effect of PKU, or a consequence of the controlled diet consumed by PKU patients, is still unknown. We believe that further studies exploring the nutritional quality of PKU diets and its effects on gut microbial ecology and health, in a larger number of patients and over longer periods, will help elucidate this question.

## Supporting Information

S1 Table Summary of clinical characteristics of patients with phenylketonuria and controls and Good’s sequence coverage. (DOC)

S2 Table Comparison of daily aminoacid intake. (DOCX)

S3 Table Comparison of daily intake for minerals. (DOCX)
