# Supplementary material for: Phenylketonuria and Gut Microbiota: A Controlled Study Based on Next-Generation Sequencing
Source: PLoS One. 2016 Jun 23;11(6):e0157513. doi: 10.1371/journal.pone.0157513 (PMC4918959; doi:10.1371/journal.pone.0157513)
Supplement: S1 Table — (DOC) [file pone.0157513.s001.doc]

**Table S1.** Summary of clinical characteristics of patients with Phenylketonuria and controls and Good’s sequence coverage.

| **Subjects** | **Sex** | **Age (y)** | **Weight (kg)** | **Height (cm)** | **BMI** | **BMI**  **Z-score*** | **Age at diagnosis (days)** | **Plasma Phe (µmol/L)^a^** | **Plasma Tyr (µmol/L) ^b^** | **Daily Intake of Protein from Protein Substitute (%)** | **Type of Protein Substitute** | **Good’s sequence Coverage** |
| --- | --- | --- | --- | --- | --- | --- | --- | --- | --- | --- | --- | --- |
| PKU 1 | Male | 0.75 | 7.84 | 67 | 17.46 | 0.21 | 3 | 98.6 | 71.2 | 47.24 | PKU Med A^TM^ | 0.999 |
| PKU 2 | Male | 1.58 | 10.38 | 80 | 16.22 | 4,33 | 22 | 290.4 | 30.91 | 61.89 | PKU Med A^TM^ | 0.999 |
| PKU 3 | Male | 1.25 | 12.43 | 83 | 17.83 | 2.02 | 8 | 129.47 | 63.48 | 95.29 | PKU Med B^TM^ | 0.999 |
| PKU 4 | Male | 2.67 | 15.01 | 94 | 16.99 | -1.28 | 60 | 189.97 | 88.32 | 72.33 | PKU Med B^TM^ | 0.999 |
| PKU 5^c^ | Male | 0.25 | 5.06 | 60 | 14.06 | -9.34 | 30 | 289.8 | 100.46 | 89.68 | PKU Med B^TM^ | 0.999 |
| PKU 6 | Male | 6.42 | 39.20 | 132 | 22.33 | 2.35 | 100 | 605 | 117.5 | 88.61 | PKU Med B^TM^ | 0.997 |
| PKU 7 | Female | 15.00 | 56.50 | 148 | 25.62 | 1.3 | 60 | 571.1 | 46.36 | 77.30 | PKU Med C Plus^TM^ | 0.997 |
| PKU 8 | Female | 6.00 | 21.80 | 112 | 17.38 | 0.88 | 30 | 284.35 | 34.22 | 51.97 | PKU Med B^TM^ | 0.999 |
| C 1^c^ | Male | 0.67 | 10.00 | 90 | 12.35 | -3.56 | - | NA | NA | NA | - | 1.000 |
| C 2 | Male | 3.67 | 23.00 | 91 | 27.77 | 7.27 | - | NA | NA | NA | - | 0.998 |
| C 3 | Male | 5.83 | 21.80 | 119 | 15.39 | -0.54 | - | NA | NA | NA | - | 0.996 |
| C 4 | Male | 15.17 | 64.20 | 183 | 19.17 | -0.3 | - | NA | NA | NA | - | 0.998 |
| C 5 | Female | 1.67 | 12.00 | 89 | 15.15 | -0.38 | - | NA | NA | NA | - | 1.000 |
| C 6 | Female | 5.25 | 23.00 | 120 | 15.97 | 0.57 | - | NA | NA | NA | - | 0.985 |
| C 7 | Female | 5.67 | 18.50 | 115 | 13.99 | -1.53 | - | NA | NA | NA | - | 0.998 |
| C 8 | Female | 4.00 | 13.00 | 99 | 13.26 | 0.94 | - | NA | NA | NA | - | 0.997 |
| C 9 | Female | 17.17 | 54.00 | 155 | 22.48 | 0.35 | - | NA | NA | NA | - | 0.997 |
| C 10 | Female | 1.50 | 10.50 | 89 | 13.26 | -2.12 | - | NA | NA | NA | - | 0.998 |

NA, not available; Phe, phenylalanine; Tyr, tyrosine

^a^ Blood Phe reference values: 2-18y = 26-91 µmol/L; >18y= 42-74 µmol/L.

^b^ Blood Tyr reference values: 2-18y=24-115 µmol/L; >18y= 26-78 µmol/L.

^c^ Patient and Control in breastfeeding during the study.

* Evaluated as percentile of BMI for age according WHO.
