# Supplementary material for: Phenylketonuria and Gut Microbiota: A Controlled Study Based on Next-Generation Sequencing
Source: PLoS One. 2016 Jun 23;11(6):e0157513. doi: 10.1371/journal.pone.0157513 (PMC4918959; doi:10.1371/journal.pone.0157513)
Supplement: S2 Table — (DOCX) [file pone.0157513.s002.docx]

Table S2 - Comparison of daily aminoacid intake

| Daily intake (mg/day) | | | | | | | | |  |
| --- | --- | --- | --- | --- | --- | --- | --- | --- | --- |
| *Amino acids* | ***Total (mg/day)*** | | | ***From Metabolic Formula (mg/day)*** | | | ***From diet (mg/day)*** | |  |
|  | PKU Patients (n= 8) Mean ± SEM | Controls (n=10) Mean ± SEM | **p** value | PKU Patients (n=8)  Mean ± SEM | Controls (n=10) Mean ± SEM | **p** value | PKU Patients (n=8)  Mean ± SEM | Controls (n=10) Mean ± SEM | **p** value |
| Arginine | 4458.75 ± 779.64 | 3544 ± 251.74 | 0.328 | 3858.75 ± 838.39 | - | - | 658.71 ± 165.39 | 3544 ± 251.74 | **0.001** |
| Cystine | 2755 ± 540.24 | 785 ± 58.54 | 0.062 | 2605 ± 562.39 | - | - | 171.42 ± 44.69 | 785 ± 58.54 | **0.001** |
| Glutamic acid | 13686.5 ± 2212.4 | 10550 ±66.78 | **0.001** | 11590 ± 2545.69 | - | - | 2391.42 ± 799.71 | 10550 ± 665.78 | **0.001** |
| Glycine | 3846.25 ± 585.08 | 3111 ± 235.73 | **0.010** | 3463.75 ± 651 | - | - | 437.14 ± 100.49 | 3111 ± 235.73 | **0.001** |
| Histidine | 2903.75 ± 533 | 1811± 106.36 | 0.155 | 2646.25 ± 569.93 | - | - | 294.28 ± 84.65 | 1811 ± 106.36 | **0.001** |
| Isoleucine | 5711.25 ± 1093 | 2748 ± 147.1 | 0.062 | 5302.5 ± 1150.06 | - | - | 467.14 ± 120.88 | 2748 ± 147.12 | **0.001** |
| Leucine | 9371.25 ± 1432.3 | 4773 ± 246.65 | **0.011** | 8661.25 ± 1589.62 | - | - | 811.42 ± 226.45 | 4773 ± 246.65 | **0.001** |
| Lysine | 7896.25 ± 15667 | 4434 ± 276 | 0.131 | 7442.5 ± 1625.13 | - | - | 518.57 ± 159.30 | 4434 ± 275.98 | **0.001** |
| Methionine | 1990 ± 378.6 | 1458 ± 86.74 | 0.374 | 1843.75 ± 397.01 | - | - | 167.14 ± 40.33 | 1458 ± 86.74 | **0.001** |
| Phenylalanine | 512.25 ± 138.23 | 1872 ± 284.50 | **0.004** | - | - | - | 512.25 ± 138.23 | 1872 ± 284.49 | **0.004** |
| Proline | 8560 ± 1604.45 | 3638 ± 221.38 | **0.010** | 7885 ± 1711.24 | - | - | 771.42 ± 253.14 | 3638 ± 221.38 | **0.001** |
| Serine | 6290 ± 1189.74 | 2492 ± 134.12 | **0.011** | 5806.25 ± 1258.25 | - | - | 552.85 ± 149.51 | 2492 ± 134.12 | **0.001** |
| Tryptophan | 2131.25 ± 415.23 | 554 ± 39.27 | **0.001** | 1998.75 ± 434 | - | - | 151.42 ± 38.75 | 554 ± 39.27 | **0.001** |
| Tyrosine | 7042.5 ± 1409.09 | 1983 ± 108.32 | **0.001** | 6788.75 ± 1441.21 | - | - | 290 ± 71.01 | 1983 ± 108.32 | **0.001** |
| Valine | 5365 ± 1319.8 | 3101 ± 147.66 | 0.374 | 4861 ± 1365.07 | - | - | 575.71 ± 144.89 | 3101± 147.66 | **0.001** |

PKU, phenylketonuria.

Numeric variables were summarized as means ± SEM and compared using the Mann-Whitney *U* test. Significant *p*-values (< 0.05) highlighted in bold.
