# Supplementary material for: Phenylketonuria and Gut Microbiota: A Controlled Study Based on Next-Generation Sequencing
Source: PLoS One. 2016 Jun 23;11(6):e0157513. doi: 10.1371/journal.pone.0157513 (PMC4918959; doi:10.1371/journal.pone.0157513)
Supplement: S3 Table — (DOCX) [file pone.0157513.s003.docx]

Table S3 - Comparison of daily intake for minerals

| Daily intake (mg/day) | | | | | | | | |  |
| --- | --- | --- | --- | --- | --- | --- | --- | --- | --- |
| *Minerals* | ***Total (mg/day)*** | | | ***From Metabolic Formula (mg/day)*** | | | ***From diet (mg/day)*** | |  |
|  | PKU Patients (n= 8) Mean ± SEM | Controls (n=10) Mean ± SEM | **p** value | PKU Patients (n=8)  Mean ± SEM | Controls (n=10) Mean ± SEM | **p** value | PKU Patients (n=8)  Mean ± SEM | Controls (n=10) Mean ± SEM | **p** value |
| Calcium | 2015.93 ± 430.35 | 546.03± 117.30 | **0.001** | 1818.82 ± 420.13 | - | - | 197.10 ± 28.03 | 546.03 ± 117.30 | **0.013** |
| Iron | 32.64 ± 6.80 | 9.04 ± 0.83 | **0.001** | 26.64 ± 6.68 | - | - | 5.99 ± 1.28 | 9.04 ± 0.83 | 0.076 |
| Magnesium | 277.21 ± 69.37 | 162.73 ± 9.94 | 0.248 | 165.80± 74.99 | - | - | 111.40 ± 28.93 | 162.73 ± 9.94 | 0.110 |
| Manganese | 3.73 ± 1.02 | 1.32 ± 0.20 | **0.023** | 2.52± 1.25 | - | - | 1.52 ± 0.37 | 1.32 ± 0.20 | 0.689 |
| Phosphorus | 1343.48 ± 252.74 | 769.44 ± 65.32 | **0.041** | 1075.82 ± 251.21 | - | - | 267.66 ± 65.09 | 769.44 ± 65.32 | **0.001** |
| Potassium | 259.46 ± 457.14 | 1749.44± 177.15 | 0.183 | 1155.95 ± 305.77 | - | - | 1353.51 ± 275.30 | 1749.44 ± 177.15 | 0.328 |
| Selenium | 20.19 ± 9.94 | 82.88 ± 7.37 | **0.003** | 0.017 ± 0.017 | - | - | 20.18 ± 9.94 | 82.88 ± 7.37 | **0.003** |
| Sodium | 824.92 ± 147.82 | 1107.32 ± 105.98 | 0.110 | 103.09 ± 55.12 | - | - | 721.83 ± 157.44 | 1107.32 ± 105.98 | 0.051 |
| Zinc | 21.39 ± 4.30 | 11 ± 0.78 | **0.016** | 19.58 ± 4.43 | - | - | 1.81 ± 0.44 | 11 ± 0.78 | **0.001** |

PKU, phenylketonuria.

Numeric variables were summarized as means ± SEM and compared using the Mann-Whitney *U* test. Significant *p*-values (< 0.05) highlighted in bold.
